# The utilization of humanized mouse models for the study of human retroviral infections

**DOI:** 10.1186/1742-4690-6-76

**Published:** 2009-08-12

**Authors:** Rachel Van Duyne, Caitlin Pedati, Irene Guendel, Lawrence Carpio, Kylene Kehn-Hall, Mohammed Saifuddin, Fatah Kashanchi

**Affiliations:** 1 Microbiology, Immunology, and Tropical Medicine Program, The George Washington University School of Medicine, Washington, DC 20037, USA; 2 Department of Microbiology, Immunology, and Tropical Medicine, The George Washington University School of Medicine, Washington, DC 20037, USA; 3 CONRAD, Eastern Virginia Medical School, 1911 Fort Myer Drive, Suite 900, Arlington, VA 22209, USA

## Abstract

The development of novel techniques and systems to study human infectious diseases in both an *in vitro *and *in vivo *settings is always in high demand. Ideally, small animal models are the most efficient method of studying human afflictions. This is especially evident in the study of the human retroviruses, HIV-1 and HTLV-1, in that current simian animal models, though robust, are often expensive and difficult to maintain. Over the past two decades, the construction of humanized animal models through the transplantation and engraftment of human tissues or progenitor cells into immunocompromised mouse strains has allowed for the development of a reconstituted human tissue scaffold in a small animal system. The utilization of small animal models for retroviral studies required expansion of the early CB-17 *scid/scid *mouse resulting in animals demonstrating improved engraftment efficiency and infectivity. The implantation of uneducated human immune cells and associated tissue provided the basis for the SCID-hu Thy/Liv and hu-PBL-SCID models. Engraftment efficiency of these tissues was further improved through the integration of the non-obese diabetic (NOD) mutation leading to the creation of NODSCID, NOD/Shi-*scid IL*2*r*γ^-/-^, and NOD/SCID β2-microglobulin^null ^animals. Further efforts at minimizing the response of the innate murine immune system produced the Rag2^-/-^γ_c_^-/- ^model which marked an important advancement in the use of human CD34+ hematopoietic stem cells. Together, these animal models have revolutionized the investigation of retroviral infections *in vivo*.

## HIV-1 Pathogenesis

The HIV-1 virus is the etiologic agent of AIDS (Acquired Immunodeficiency Syndrome) and a life-long infection results in the destruction of lymphocytes, rendering the host immunocompromised [1,2]. The development of AIDS in HIV-1 infected individuals has been defined as a result of a combination of two different types of infections characterized by an acute phase where the virus can rapidly deplete CD4+ T cells and a chronic phase where the damaged immune system gradually loses all functionality [3-5]. Though the primary target is CD4+ T cells, the HIV-1 virus can also infect both monocytes/macrophages and dendritic cells (DCs), however, cellular tropism of the virus is determined by the expression of the cell surface receptor CD4 and the coreceptors CCR5 and CXCR4. Genetic variability in the expression of these cell surface markers can lead to differences in susceptibility by so-called R5 viruses which recognize CCR5, R5X4 viruses which recognize both CCR5 and CXCR4, and X4 viruses which recognize only CXCR4 [6-8]. The activity and longevity of the integrated HIV-1 provirus can be directly correlated to both the activation state as well as the survival of the cell. This phenomenon results in dramatically different viral pathogenicity in activated as compared to both resting and quiescent CD4+ T cells [3,9,10]. Primary HIV-1 infection is asymptomatic during the first two weeks after exposure to the virus; however, acute HIV-1 infection is evident by a dramatic burst of viral replication correlating with infection of activated T cells. This initial infection and high viral replication efficiency result in a high titer of virus present in the plasma of infected individuals that gradually drops off as the infection induces a cytopathic effect on the T cells after approximately nine weeks post infection. This acute viremia is also correlated with an active host immune response against the infection in the form of cytotoxic T lymphocyte (CTLs) CD8+ cells that recognize HIV-1 infected cells and induce cell death [11-13]. This CD8+ CTL response correlates with the production of HIV-1 neutralizing antibodies or seroconversion of the patient. An additional population of CD4+ T cells can be classified as resting or permissive where cellular replication is restricted at several different steps; however, there exists enough stimulatory signals to push the cell into the G_1 _phase of the cell cycle. In HIV-1 positive individuals, the resting CD4+ T cells contain HIV-1 DNA in a linear form (in the cytoplasm of the cell) representing an inducible viral population that can be properly integrated upon the correct stimulation. Despite the cytoplasmic localization of the majority of viral DNA, low levels of integrated HIV-1 can be isolated from a small subset of the resting T-cell population which is most likely due to infected, activated CD4+ T cells that have reverted back to a resting state, a commonly seen phenomenon important for the establishment of immunologic memory [14,15]. Similarly, infected quiescent or refractory CD4+ T cells also exhibit viral replication restrictions where the provirus exists integrated in the genome in a silent or latent state [15-18]. The establishment of transcriptionally silent provirus does not occur only in this subset of T cells; indeed, actively dividing T cells can contain viral reservoirs as latency can be an intrinsic property of the virus [19]. It is assumed that the provirus is established in these cells during normal progression through the cell cycle and in response to the infection to avoid cytopathicity and immune clearance. After the reverse transcription step has been completed, the cell establishes itself at G_0_, blocking further progression [3,15,18]. This establishment of a latent population of cells containing integrated provirus signifies the clinical latency period of infection, where the maintenance of T cell homeostasis and low viral loads occur until the terminal stages of infection and progression to disease [15,18,20,21].

The fidelity of the HIV-1 RT as well as the rapid viral replication rate contribute to the diversity of the viral progeny. In an active infection 10^9^-10^10 ^virions are produced per day, and during each viral replication cycle there is a mutation rate of approximately 3 × 10^-5 ^nucleotides due primarily to a "slippery" RT [22,23]. The introduction of multiple point mutations in the viral genome results in many different strains of virus within an infected individual, as well as the possibility of one cell being infected by different strains, leading to recombination events. Additionally, the genomic variability leads to differences in protein sequence and structure, resulting in difficulties in developing antiretroviral drugs against the viral integrase, protease, and RT. This results in the appearance of drug-resistant HIV-1 variants in the face of antiretroviral therapies. This necessitates a cocktail of antiretroviral drugs known as HAART (highly active antiretroviral therapy) as the primary treatment for HIV-1 infected individuals who need to be constantly evaluated for treatment effectiveness against the viral strains present [24-29].

In addition to the primary infection of susceptible populations of CD4+ T cells and monocytes/macrophages DCs can also support the integration of proviral DNA [3,30]. Tissue macrophages are infected primarily through the CCR5 coreceptor, and individuals that lack CCR5 are highly resistant to infection, irrespective of CD4+ T cell infection [31-34]. Infection of tissue macrophages assists in the progressive infection of CD4+ T cells due to interactions of the HIV-1 viral protein Nef through stimulation of the CD40 receptor and activation of the NF-κB pathway [35]. Subsequent secreted proteins increase the expression of stimulatory receptors on B cells, which then interact with corresponding ligands on CD4+ T cells, allowing for either viral entry and the expression of viral proteins or the productive infection of susceptible CD4+ T cells [35].

The loss of CD4+ T cells in HIV-1 infected individuals leaves the host susceptible to opportunistic infections, many of which are normally blocked through mucosal barriers and innate immunity. The infection of the gut-associated lymphoid tissue (GALT) of the HIV-1 infected gastrointestinal (GI) tract and the pathogenesis surrounding this manifestation are termed HIV enteropathy [36-40]. Viral replication within the GALT tissue is compartmentalized with different anatomical areas of the gut exhibiting higher levels of infected cells in one site than others, i.e. esophagus, stomach, duodenum and colorectum [41]. This is due largely to the wide range of distribution and composition of lymphoid tissues in the gut, including Peyer's patches in the small intestine, lymphoid follicles in the large intestine and rectum, and a majority of CD8+ T cells in the intraepithelium of the small intestine [41]. This situation allows for the selection of various HIV-1 susceptible cell types within different areas of the GALT. The HIV-1 induced local activation and inflammation of the GI immune system result in the recruitment and infiltration of CD4+ T cells and CD8+ T cells to the mucosal tissues [38]. Indeed in HIV-1 infected individuals, there is an increase in the proinflammatory lymphocyte response as well as an absence of CCR5+ CD4+ T cells within the GI tract during the acute stage of infection. Rapid elimination of CD4+T cells associated with structural damage of the gut is thought to cause leakage of bacterial pathogens/products into the blood stream resulting in hyperimmune activation, the hallmark of immunopathogenesis of HIV disease [42]. CD4+ T cells in the GI tract are 10-fold more likely to be infected by HIV-1 than those in the peripheral blood; however, the predominance of HIV-1 specific CD8+ T cells in the GI tract is comparable to the CD4+ levels observed in peripheral blood [43-45]. The induction of a mucosal humoral immune response through activation of a functional HIV-1 specific T-cell response may help to control viral replication and inhibit viral spread within the GI tract.

## Comparison of animal models for the study of retroviral infection

The identification of HIV-1 as the causative agent of AIDS was followed only a year later by the recruitment of chimpanzees for the purpose of *in vivo *research into the disease and its associated pathogenesis, treatment, and prevention [46]. Chimpanzees represented a logical and ideal starting animal model because of their documented DNA homology with humans; the two species share between 97 and 98% of their genomes. However, on a practical level, this animal was also recognized as an endangered species in certain areas; and despite genetic similarities, there are also many differences that affect immune responses and clinical manifestations of infection with human viruses, such as HIV-1 [46,47].

Early experiments in the 1980s utilizing chimpanzees demonstrated a series of important insights into HIV-1 infection, including the ability to be transmitted through blood and vaginal secretions [46]. These investigations were able to establish an HIV-1 infection of HIV-1 in chimpanzees with successful viral entry, expression, subsequent productive viral replication and even IgG immune response mimicking human conditions. However, important differences in cell-mediated immune responses began to emerge, especially in the case of the studies by Zarling *et al*. where they observed that CTLs that developed in humans and played an important role in pathogenesis were not present in chimpanzees [48]. Chimpanzees were also not developing the same markers of disease as humans, such as increases in β2 microglobulin, TNF-α, and IL-6. Attention shifted to other options including the use of HIV-2 and Simian Immunodeficiency Virus (SIV) as infection models. HIV-2 proved successful in infecting cynomologus macaques while SIV was useful for investigating clinical progression, particularly in juvenile macaques, of immunodeficiency as it compared to the disease in humans [49-51]. However both of these systems have limitations including differences in the natural progression of disease as well as challenges in accurately targeting therapeutic interventions, in addition to the high cost of animals. The combination of the HIV-1 envelope gene with the naturally occurring lentivirus in primates, SIV, produced a chimeric virus known as SHIV [52]. SHIV models in rhesus and pigtail macaques have provided some success as surrogates for HIV-1 infection in humans. However, a major difference remains, the development of AIDS, occurring in this primate model within about 2–6 months period as opposed to the often longer latency observed in humans. Therefore this SHIV model is considered a useful representation of acute infection that progresses rapidly but is not necessarily an accurate reflection of the insidious HIV-1 infection and disease course. Some SIV strains such as SIVmac251 do in fact demonstrate more of a chronic infection and have found some success in efforts aimed at vaccine development, though some differences with HIV-1 still exist with regard to pathogenesis. Recent data show that chimpanzees infected with SIVcpz are able to develop an immunopathology similar to human AIDS [53] suggesting that this model holds further utility.

Despite the usefulness of non-human primates for investigations of human retroviruses, the difficulties encountered with respect to ethical, financial, and immunological challenges have led quickly to the exploration of smaller animal models (Table 1). One such model utilizing feline immunodeficiency virus (FIV) infection has provided limited insight for comparison to human disease, though this model has shown some promise vaccine development efforts and also in relevance for to human neuropathy related to HIV infection [54,55]. Rats have also been utilized for pharmacological research as well as HIV-1 associated dementia [47]. Transgenic animals, both rat and mouse, have also demonstrated value especially for investigations concerning entry or the effects of viral integration on specific tissues [47]. However, transgenic animals are limited in the ability to study therapeutics or vaccines since viral replication and proliferation are not fully achieved in these models [47]. In particular, the major impairment in the transgenic rat models occurs at the level of viral gene expression and maturation of viral particles [56,57]. While it is possible to infect these animal models with HIV-1, problems arise in the later stages of the viral life cycle resulting in an inability to sustain viral production. Although these transgenic models could mimic the early events in viral replication, a significant block is encountered at the point of integration, ultimately creating a limited picture of productive systemic infection [58]. Recent developments have shown that murine models (e.g. humanized mice) have become increasingly desirable for retroviral infection studies. Mice represent an ideal research option not only for their relatively low cost and ease of access, but also because of the ever increasing ability to manipulate the mouse genome in order to more accurately reflect what is happening in human infection at both the molecular and clinical levels [47]. These murine models are continuing to evolve, and new approaches are being developed for establishing an accurate picture of human retroviral infection and for allowing relevant investigation of therapeutic and preventive options.

**Table 1:** Comparison of Animal models for the Investigation of Retroviral Infections

**Type of Model**	**Viral Infection**	**Method of Infection**	**Advantage**	**Disadvantage**
**Non-Human Primates** (chimpanzees, rhesus, pigtail, cynomologus ymacaques, etc.)	• HIV-1	• IV	• Useful for vaccine and therapeutic studies	• SIV/SHIV are surrogates for HIV infection
	• HIV-2	• Vaginal	• Genetic similarities between species	• Differences in time course of disease
	• SIV	• Rectal		• Differences in molecular and cellular markers
	• SHIV			• Significant cost and ethical concerns

**Feline**	• FIV	• IV	• Insight into neurological AIDS complications	• Strictly surrogate model
		• Vaginal	• Pharmacological and vaccine studies	
		• Rectal		

**Transgenic Mice/Rats**	• HIV-1	• IV	• Cost and accessibility	• Lack of viral replication and proliferation
			• Manipulation of genome	
	• None	• Transgenic insertion of HIV genes	• Fusion and entry	
			• Effect of virus on different tissues	

**Humanized Mice**	• HIV-1	• IV	• Cost and accessibility	• Further characterization of pathogenesis and continued evolution of model expected
		• IP	• Manipulation of genome	
		• Vaginal	• Creation of human immune system scaffold for proliferating virus	
			• Mucosal infections	
		• Rectal	• Vaccine and therapeutics at varying stages of viral life cycle	
		• Thy/Liv		

## A brief history of humanized mouse models

**Figure 1:**
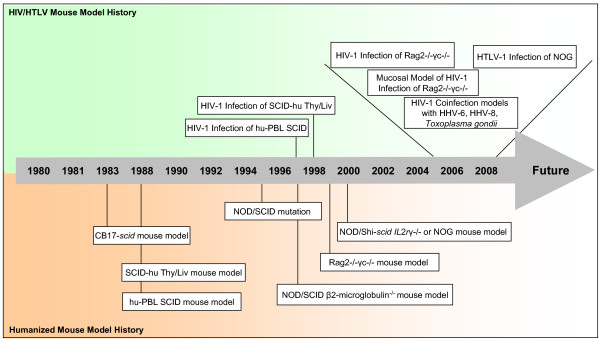
**A timeline of humanized mouse model development and retroviral research**. A highlight of the noteworthy events of humanized mouse model system development over the past 30 years. The bottom half of the timeline denotes the emergence of key humanized mouse models. The top half of the timeline denotes the application of the models to HIV-1 and HTLV-1 research. The area from 2005 to 2009 has been expanded to show the increase in retroviral development within a short time period.

The first humanized mouse model to be developed was in 1983 by Bosma *et al*. through the discovery of the *scid *mutation in CB-17 *scid/scid *(SCID) mice [59]. These mice contained an autosomal recessive mutation in the *prkdc *(protein kinase, DNA activated, catalytic polypeptide) gene resulting in a deficiency in mature T and B lymphocytes. This mutation resulted in the ability of these mice to accept foreign tissues, therefore allowing the engraftment of human cells and/or tissues. This model represents the landmark experiment that sparked further development of humanized mice for the study of human hematopoiesis. In the late 1980's both the SCID-hu Thy/Liv [60,61] and the hu-PBL-SCID [62,63] mouse models were developed, where human thymus and liver and human peripheral blood mononuclear cells (PBMCs), respectively, were successfully engrafted. In 1995, the SCID mutation that had been utilized in other models was crossed with the non-obese diabetic (NOD) mouse model resulting in an animal (NOD-SCID) that demonstrated a marked increase in engraftment potential. These animals could accept the xenotransplantation of blood cells forming fetal liver, bone, thymus, and lymphoid cells [60,61,64-67]. Further adjustments have been made to this NOD/SCID model over time in order to continue to increase the extent and efficiency of humanization that could be achieved, resulting in the development of the NOD/SCID β2-microglobulin^null ^and the NOD/SCID *IL*2*r*γ^null ^mouse models [68,69]. Recently, a mouse model defective in common γ chain (γ_c_) receptor for IL-2, IL-7, IL-15 and other cytokines, was made from the recombinase activating gene (Rag) knockout mice [70-73] as well as from the NOD-SCID mouse [71]. These Rag^-/-^γ_c_^-/- ^and NOD-SCID γ_c_^null ^(NOG) mice have no functional T, B, or NK cell activity in addition to being superior to the SCID mice, due to the lack of a leaky mutation. All of these mouse models have developed over time to various degrees of accuracy and efficiency of xenotransplantation of human cells/tissues as well as the development of a functioning human immune system. Due to differences in experimental approach and limitations on life-span, each mouse strain is suitable for a specific kind of experimental model. Here, we focus on the development of each of these models for the study of human retroviral infection, i.e., with HIV-1 and HTLV-1. The comparison of all of these models in historical context, as illustrated in Figure 1, provides extensive background information and reviews the recent literature. In addition, the implication of these humanized mouse models in the study of retroviral coinfections with other pathogens will be addressed.

## Graft vs. Host disease in humanized mouse models

An inherent problem associated with the engraftment of any foreign tissue into another host is the risk of incompatibility, either rejection of the graft by the host or graft vs. host disease (GVHD). GVHD is an interesting and especially relevant syndrome that is often observed in organ and bone marrow transplants when functional immune cells in the transplanted tissue or fluid recognize the host cells and tissue as foreign and subsequently initiate an immunologic response against the host. This response quickly spreads to become an established systemic attack and results in the death of the host. In the context of xenografted small animals, how is it that these humanized mice can support and establish a functioning human immune system without exhibiting any GVHD symptoms? One possible answer is found in the Thy/Liv model which has proven particularly useful in preventing GVHD due to the complete exclusion of mature CD3+ T cells, a phenomenon that can be mimicked clinically with some success. Additionally, the presence of the fetal thy/liv organ allows for innate maturation of human CD4+ and CD8+ T cells in the context of the animal's own immune system.

In general, the proliferation of human cells in these humanized mouse models is clearly evident; however, the functionality of the system is under scrutiny. Uittenbogaart *et al*. have shown that the maturation of engrafted human T cells occurs within the microenvironment of the SCID mouse; however, the possibility of phenotypic changes, especially on cell-surface markers is evident [74]. It is possible that these animals may actually exhibit an atypical GVH reaction, where the xenografted human T cells become anergic within the mouse [75]. The CD4+ and CD8+ populations of T cells in particular, exhibit anergy in that they are not activated to secrete cytokines after stimulation with CD3; however, when grown *in vitro*, the chimeric CD4+ cells were able to display anti-SCID mouse reactivity [75]. These data suggest that although the SCID mouse is able to support a human T cell system the immune system may not always be properly functional. It has been proposed that up to three weeks post engraftment, a majority of the injected human cells will survive, proliferate, and mature; however, after this time, anti-mouse-reactive clones that are selected for and the engrafted immune system becomes nonfunctional [63]. Finally, exploring the apparent contradictory lack of GVDH in these model systems, it is important to note that GVHD typically refers to events associated with allogenic grafts; the syndrome is not as well defined, understood, or quantified in xenogenic grafts.

## Humanized murine models of HIV-1 infection

### SCID-hu Thy/Liv Mice and HIV-1

The discovery of the severe combined immunodeficiency mutation (*scid*) in the CB17-scid/scid mice strain in 1983 gave rise to the development of the SCID-hu Thy/Liv model, the first reported attempt of murine humanization in 1988 [61]. The now well characterized SCID-hu Thy/Liv model has been described as a valuable *in vivo *system for the developing field of translational research due to its multi-functionality in areas of experimental research [75]. The SCID-hu Thy/Liv model is a heterochimeric small animal system where severe combined immunodeficient CB17-scid (SCID) mice with a phenotype characterized by the absence of mature B, T cells and radiation sensitivity [59,76] are transplanted with human fetal thymus and liver tissues under the kidney capsule. The co-implanted human thymus and liver tissues fuse in the formation of a conjoint organ (Thy/Liv) that continuously produces long-term (6 months to ≥ 12 months) human hematopoietic CD34+ progenitor stem cells as well as normal mature human lymphocytes with a majority (>70%) of CD4/CD8 double-positive (DP), CD4+ and CD8+ single-positive (SP), and double-negative (DN) T cells [77] (Table 2). After implantation, the SCID-hu Thy/Liv mice develop peripheral blood lymphocytes (PBL) consisting mostly of naive CD4+ or CD8+ SP T cells that display migration from the human thymus and liver engraftment to the periphery in a time lapse of 3–4 weeks post-surgery; however, there is no significant systemic repopulation of human T cells and practically no human B cells, monocytes, macrophages, or DCs [78]. The SCID-hu Thy/Liv mice have been appropriately used for tissue transplants, human hematopoiesis analysis and the study of HIV-1 infection pathophysiology, as well as the *in vivo *efficacy of immunomodulatory, drug and gene therapies [60,79-81]. Overcoming some challenges of these reconstituted SCID-hu mice, the model allows for the production of single-donor large cohorts that increase statistical significance of comparative pre-clinical drug trials [82-84].

**Table 2:** Defining Characteristics of Humanized Mouse Models

**Model**	**Human Cells Engrafted**	**Irradiation**	**Demonstrated Human Cells**	**Humanized Tissues**	**Length of Detection**
SCID-hu Thy/Liv	Fetal thymus and liver	No	D4/CD8 DP, SP, DN, T cells in peripheral blood	Peripheral blood, fused thy/liv organ	6 to ≥ 12 months
hu-PBL SCID	IP PBMCs	No	CD4/CD8 SP T cells, CD3+ T cells, monocytes, NK cells, and B cells	Lymph nodes, spleen, liver, bone marrow	6 months
NOD SCID BLT	Fetal thymus and liver, fetal liver tissue-derived CD34+ stem cells	Yes	Mature T and B lymphocytes, monocytes, macrophages, and dendritic cells	Peripheral blood, liver, lung, vagina, rectum, and GALT	22 weeks
NOD SCID IL2r γ^-/-^	CD34+ human cord blood	Yes/No	Myelomonocytes, dendritic cells, erythrocytes, platelets, and lymphocytes	Peripheral blood, spleen, and bone marrow	> 300 days
Rag2^-/-^γc^-/-^	CD34+ human cord blood	Yes	Dendritic, T, and B cells	Peripheral blood, liver, spleen, bone marrow, vagina, GALT	190 days
NOD SCID β2m	Transformed HTLV-1 cell lines, PBMCs from HTLV-1 infected patients	Yes/No	CD45+, CD3+, T cells	Peripheral blood, spleen, lymph node, bone marrow	4 to 12 weeks
NOD SCID IL2rγ null ("NOG")	Transformed HTLV-1 cell lines, PBMCs from HTLV-1 infected patients	No	CD4+, CD8+ T cells	Liver, spleen, lung, kidney	N/A

An intrathymic or intranodal injection of HIV-1 into the SCID-hu Thy/Liv mouse results in an infection that mimics human viral tropism; that is, preferential infection of CD4+ T cells [85]. Immunohistological staining revealed infected cells primarily in the thymus cortical regions, spreading later through the entire heterochimeric thymus as the infection progressed [77,86]. Interestingly, only primary isolates of HIV-1 (JR-CSF) derived from patients were permissive for viral replication in the SCID-hu Thy/Liv mouse as compared to a lab strain (IIIb) which produced no detectable viral RNA. After the intravenous or intraorgan infection with HIV-1, only human cells were infected and from these, only CD4+ T and myelomonocytic cells. Initial HIV-1 infections of SCID-hu Thy/Liv animals resulted in a near-eradication of CD4+/CD8+ DP thymocytes and a decrease in the CD4+ SP T cell population of the human implanted tissue [77,87,88], a depletion shown to be reduced upon treatment with several anti-HIV compounds [89-93] (Table 3).

**Table 3:** Defining Characteristics of Retroviral Infection in Humanized Mouse Models

**Model**	**Strain of Virus**	**Method of Infection**	**Active Viremia (after how long)**	**Infected Tissues**	**Depletion of T Cells?**	**Neutralizing Ab?**
SCID-hu Thy/Liv	HIV-1 (R5, X4)	IV or intraorgan	Within a few weeks	CD4+ T and myelomonocytic cells	Yes	No
hu-PBL SCID	HIV-1 (R5, X4)	IP or intraorgan	Within 2 weeks	T cells, vaginal	Yes	Yes
NOD SCID BLT	HIV-1 (R5)	IP, vaginal, rectal	Within a few weeks	Vaginal, rectal, GALT	Yes	Yes
NOD SCID IL2r γ^-/-^	HIV-1 (R5, X4)	IP, IV	Within a few weeks	Peripheral blood, spleen, bone marrow, thymus, vaginal	Yes	Yes
Rag2^-/-^γc^-/-^	HIV-1 (R5, X4)	IP, vaginal, rectal	Within 2 weeks	Peripheral blood, thymic, splenic, and lymphoid tissues, vaginal and rectal mucosa	Yes	Yes
NOD SCID β2m	HTLV-1 (transformed cell lines)	IP, IV	Between 3 and 12 weeks	Peripheral blood, spleen, lymph nodes, bone marrow	N/A	N/A
NOD SCID IL2rγ null ("NOG")	HTLV-1 (transformed cell lines)	IP, IV	Within 2 weeks	Peritoneal cavity, spleen, peripheral blood	N/A	N/A

Significant disadvantages of the SCID-hu Thy/Liv, due to suboptimal conditions for the establishment of a complete human immune system *in vivo*, have propelled the development of improved models. Largely, the CB17-scid is known to exhibit high levels of innate immune and NK cell activity, and age-related spontaneous generation of mouse B and T cells that in turn lowers the levels of successful engraftment of human tissue [76,94,95]. In 1994, in an attempt to correct the low count of human PBLs, Kollman *et al*. implanted greater amounts of Thy/Liv tissue beneath both kidney capsules, in effect producing higher levels of detectable circulating human T cells and a consequent variation to this model [96]. Noteworthy, the surgical procedure for implantation of the human fetal thymus and liver tissues requires skilled researchers for co-implantation as well as systemic support of the developing organoid [77].

Additionally, this model is not an appropriate scaffold for the study of the humanized immune system or HIV-1 infection of mucosal tissues such as vaginal, rectal, or GALT largely due to the confinement of most of the engrafted human cells to the developed organoid [78]. The Thy/Liv model of HIV-1 infection still provides an appropriate platform for the evaluation of antiretroviral therapies and treatments [78]. Of particular novelty is the testing and optimization of the efficacy of such therapeutics within an intact HIV-1 infected human target organ [78]. Despite the generation of improvements as mentioned above, this humanized mouse model still maintains critical importance primarily for new antiretroviral pharmacological studies, pre-clinical testing and to a lesser extent, for the study of viral mechanisms.

### SCID-hu PBL Mice and HIV-1

The SCID-hu Thy/Liv mouse was accompanied by the development of the SCID-hu PBL (humanized-peripheral blood lymphocyte) mouse model, generated by the i.p. injection of PBMCs from healthy human adults into SCID mice [62]. These PBMCs, upon successful engraftment, tend to survive at least six months mainly in the lymph nodes, spleen, bone marrow, and genital mucosa of the SCID-hu PBL mouse [62,97,98]. These mice exhibit spontaneous secretion of human immunoglobulin (IgG) and can produce a specific human antibody response when induced with an immunization of tetanus toxoid [62]. At one day post injection, there is a large neutrophil recruitment and an induced expression of murine cytokine mRNA (IL-1 β, IL-4, IL-6, IL-10, IL-12, TNF-α and IFN-γ) that occurs in the mouse peritoneal cavity [99]. After the first three weeks of expansion of the PBL in the peritoneal cavity, the human leukocytes, specifically CD4+ or CD8+ SP T cells expressing alpha/beta T-cell receptors, begin to appear in the mouse liver and spleen [100]. In this model, the CD4+ and CD8+ cells are considered to be xenoreactive, mature, but anergic T cells. These single positive T-cells have been shown to express HLA-DR and CD45RO [100,101]. TTThe CD45RO antigen can be used as a marker for either activated or memory T-cells. There also seems to be an expansion of CD3+ T cells; however, significantly smaller numbers of human monocytes, NK cells, and B cells secrete human immunoglobulin and exhibit a secondary antibody response [102] (Table 2).

In terms of utility, the SCID-hu PBL mouse has been commonly used to study anti-HIV therapy, vaccine efficacy, as well as viral cytopathogenicity *in vivo *[101,103,104]. The SCID-hu PBL mice have been successfully implanted with CCR5- and CXCR4- tropic PBMCs-associated HIV-1 from infected individuals to an efficiency where sustained viral replication was detected by the presence of viral RNA in the plasma as well as the progressive depletion of CD4+ T cells, indicative of an acute HIV-1 infection [105]. Since SCID-hu PBL mice have a large peritoneal cavity, a large volume of CD4+T, CD8+T, and NK cells as well as complement components can exist in these mice after injection of human PBMCs and thus interaction with HIV-1 neutralizing antibodies can be tested to evaluate pre- and post- exposure protection [104] (Table 3). Administration of a high dose of the neutralizing human monoclonal antibody IgG1b12, which targets the human gp120/CD4 binding site blocked viral entry [106,107] and subsequently was able to protect the host from developing high plasma viremia [106,107]. The Rmu5.5 anti-HIV antibody was also able to protect the mice from the replication of primary isolates of HIV-1 when injected i.v. [108]. These studies demonstrated the usefulness of the SCID-hu PBL mouse as an effective model of antibody induction against HIV-1 infection; however, the studies did not show any effects of passive immunizations in mice against established HIV-1 infection.

Although the SCID-hu PBL mice have shown susceptibility to HIV-1 infection, this model does not represent a robust scaffold for genital-mucosal infection and transmission. Interestingly though, the infection of human PBLs engrafted within the vaginal tissues of these mice has been shown when the mice are pretreated with progestin to thin the vaginal epithelium [78,97,98]. This method of infection was utilized to enhance mucosal HIV-1 transmission and to evaluate the efficiency of vaginal topical microbicides.

As an attempt to improve on the existing SCID-hu PBL model, Yoshida *et al*. recognized the lack of human antigen presenting cells, such as DCs, as well as the presence of a normal human immunological lymphatic system in these mice [109]. To this end, normal human PBMCs were injected directly into the spleens of SCID mice to produce a hu-PBL-SCID-spl mouse; a hybrid of the SCID-hu PBL mouse. The mice were also implanted with human mature DCs that were treated with either inactive HIV-1 strains or control ovalbumin and then challenged with an i.p. injection of R5 HIV-1_JR-CSF_. This challenge resulted in a protective immune response and manifested the presence of neutralizing antibodies as well as other anti-HIV protective factors. These particular soluble factors were subsequently found to be produced by CD4+ T cells and are R5 viral suppressive factors [110].

## NOD-SCID models

The development of the NOD-SCID mouse model especially the CB17-*prkdc*^*scid *^mice has been described as one of the most important breakthroughs in the humanized mouse model field. The NOD-SCID mouse was created by transferring the SCID mutation into a non-obese diabetic (NOD) mouse which is often used as a model for insulin-dependent diabetes [111]. For more than a decade, NOD-SCID mice have been the "gold standard" for studies of human hematolymphoid engraftment in small animal models. The enhanced ability of NOD-SCID mice to engraft with human hematolymphoid tissues as compared with CB17-SCID mice was reported in 1995 by the Schultz group [67]. Mice in the NOD genetic background exhibit deficiencies in NK cell activity, at least partially due to impairment of the activating receptor NKG2D [112]. They are also impaired in complement activation due to C5 deficiency [113], and finally they lack LPS-induced production of IL-1 by macrophages [67]. All these features contribute to these mice showing improved engraftment of human PBMCs and hematopoietic stem cells [64,66,114,115] (Table 2). A downside to the NOD-SCID model is the tendency of the mice to develop thymic lymphomas which can compromise the life-span of the animals [111,116].

Koyanagi *et al*. described NOD-SCID as a novel immunodeficient mouse strain based its genetic background [117]. In particular, the authors described the NOD-SCID hu-PBL mouse where engraftment of human PBLs resulted in defective T, B and NK cell populations which can model a high level of HIV-1 infectable human cells. Upon infection with HIV-1, these mice exhibited high levels of viremia, as well as detectable viral RNA in infected cells, and free virions in the blood stream. This model also exhibited HIV-1 infection in vital organs such as the liver, lungs, and brain. The uniqueness of this model is derived from its lack of NK cells; therefore, the lack of innate immunity allows for the presentation of a susceptible model for the development of HIV-1 viremia as well as for multiple organ pathogenesis [117].

In the bone marrow/liver/thymus, or "BLT" mouse model, NOD-SCID mice are implanted with fetal thymic and liver organs, similar to the SCID hu Thy/Liv model [118]. The mice are then sublethally irradiated and transplanted with fetal liver tissue-derived CD34^+ ^stem cell suspension. In this model, the mice essentially undergo a bone marrow transplant to complement the human fetal thymus/liver implants [118]. This mouse model results in a large number of reconstituted human mature T and B lymphocytes, monocytes, macrophages, and DCs in lymphoid organs [118]. This model also exhibits systemic populations of a large number of human B cells, monocytes, macrophages, and DCs, in addition to the infiltration of the liver, lung, and GI tract with human immune cells (Table 2). The humanized BLT mouse is an attractive scaffold for HIV-1 research in that the robust systemic reconstitution of the mouse with human cells is possible due to the education of human T cells within the engrafted thymus, as well as the maturation of human hematopoietic cells. This system has shown functional immune responses in the form of immunoglobulin production, T cell receptor expression, and cytokine production in response to various toxins and to the xenografting itself [78]. The BLT mouse in particular contains HIV-1 susceptible populations of human cells within the GI tract as well as in the vaginal and rectal tissues [119] (Table 3). Human mucosal cells within the BLT mice are targets for mimicking HIV-1 induced CD4+ T cell depletion seen in human GALT [78,119]. In particular, the reconstituted DCs found in the gut epithelium are lineage negative, HLA-DR^bright ^CD11c^+ ^cells that are also found within the human vagina, ectocervix, endocervix, uterus, and lungs [78]. The reconstitution of the female genital tract in the BLT mice specifically provides an ideal model for the investigation of vaginal HIV-1 transmission; an infection which results in systemic dissemination of the virus in the animal.

## NOD/SCID *IL2r*γ^-/- ^mouse model and HIV-1 infection

The NOD/SCID model also served as the basis for the development of another breakthrough animal model. This time the target for mutation was the interleukin 2 receptor common gamma chain (*IL*2*r*γ^-/-^) since a defect here is responsible for the human manifestation of X-linked SCID. This mutation resulted in a significant reduction in both the innate and the adaptive immune functions and has been utilized in several different strains for the purposes of investigating the benefits of humanization [69]. In particular, the NOD/Shi-*scid IL*2*r*γ^-/- ^or NOG mouse was developed in 2000 and Ito *et al*. demonstrated its success with efficient engraftment of human hematopoietic stem cells [71]. Shultz *et al*. used a similar approach to establish the NOD/LtSz-*scid IL*2*r*γ^-/- ^mouse model [72]. These two models differ in their use of distinct NOD substrains as well as the choice of the *IL*2*r*γ^-/- ^mouse [68]. The NOG animal is the product of a cross between the NOD/Shi-*scid *mouse with an *IL*2*r*γ^-/- ^mouse that has a defect in exon 7. Conversely, Shultz's model is the result of the NOD/LtSz-*scid *animal in combination with an *IL*2*r*γ^-/- ^mouse that has a defect in exon 1 [68]. Thus far no significant differences in engraftment efficiency have been observed between the two animals, and they are considered to be comparable choices for use in investigations requiring a humanized model [68] (Table 2).

These mouse models have served as excellent tools for conducting various HIV-1 studies. This model was first shown to support human hematopoiesis by Ishikawa *et al*. who transplanted newborn NOD/SCID *IL*2*r*γ^-/- ^mice via a facial vein with purified human CD34+ cord blood cells [70]. The cells were readily reconstituted and differentiated into mature myelomonocytes, DCs, erythrocytes, platelets, and lymphocytes. This humanized model was improved upon, and it was shown that CD4+T cells in the peripheral blood, spleen, and bone marrow expressed both CXCR4 and CCR5 antigens and showed a long-lasting viremia after infection with HIV-1 viral isolates specific for both receptors [120]. The infected animals also produced both anti-HIV Env and anti-HIV Gag specific antibodies indicating a high sustained rate of viral infection. The engraftment and infection procedures employed by these studies resulted in an infection lasting only 43 days, after which the animals died; however, when the CD34+ cells were transplanted without myeloablation methods, the mice were able to survive for longer than 300 days [121] (Table 3). The establishment of a stable HIV-1 infection and a steady decline in CD4+ T cell counts resulted in one of the most efficient humanized mouse models of HIV-1 infection to date.

## Humanized Rag2^-/-^γ_c_^-/- ^Mice and HIV-1 infection

The humanized NOD-SCID models are based on the SCID mutation which can result in a leakiness marked by low level production of mouse immunoglobulins and T-cell receptors over time. Additionally, these mice have a significantly decreased viability due to the development of lethal thymic lymphomas in as little as 5 months and susceptibility to GVHD. Pertaining to HIV-1 infection, inadequate sustained hematopoietic cell populations in these mice allows for only the study of acute HIV-1 infection rather than the chronic, latent infection observed in HIV-1 infected individuals. Therefore, the development of a more stable humanized mouse model, exhibiting a functional human immune system, was needed to address the shortcomings of the hu-SCID models. This was accomplished through the development of the Rag2^-/-^γ_c_^-/- ^mice which are completely devoid of all T, B, and NK cells [122,123]. These mutant mice were created by crossing homozygous recombinase activating gene 2 (Rag2) knockout mice with homozygous common cytokine receptor γ chain (γc) knockouts [122,123]. The Rag2 mutation results in the lack of maturation of thymus-derived T cells and peripheral B cells where the γc mutation results in the lack of the functional subunit of the interleukin-2 (IL-2), IL-4, IL-7, IL-9 and IL-15 receptors, preventing the development of lymphocytes and NK cells [122,123] (Table 2). The Rag2 knockout is not a leaky mutation; it does not result in spontaneously forming tumors; nor does it confer radiation-sensitivity to the mice as the SCID mutation does. Therefore, the Rag2^-/-^γ_c_^-/- ^mouse may be an ideal scaffold for repopulation of the animal with human hematopoietic cells.

A significant advance in the humanized mouse model field was marked by the successful xenotransplantation of immunodeficient mice with human CD34+ hematopoietic stem cells (HSC). Reconstitution of human immune cells in the Rag2^-/-^γ_c_^-/- ^model and the development of human adaptive immunity has been shown by Traggiai *et al*. [73]. BALB/c Rag2^-/-^γ_c_^-/- ^neonates were sublethally irradiated, injected intrahepatically (i.h.) with CD34+ human cord blood stem cells 4–12 hours post irradiation, and allowed to reconstitute for a period of 26 weeks. Transplanted mice exhibited lymph node development at 8 weeks of age as well as the presentation of CD45+ human hematopoietic cells. The transplanted mice also developed human DC, T, and B cells, and engrafted human cells were found in the bone marrow and spleen. The investigators also showed that the engraftment was sufficient to stimulate a human immune response when exposed to tetanus toxins and Epstein-Barr virus. This was the first humanized mouse model to show any kind of normal human cytotoxic immune response. Gimeno *et al*. utilized the same mouse strain and a similar set of experiments to model the knockdown of tumor suppressor genes (i.e. p53) and monitor the development of hematopoietic cells *in vivo *[124]. Here, Rag2^/-^γ_c_^-/- ^neonates were sublethally irradiated, injected i.p. with CD34+ human cells isolated from fetal liver and allowed to reconstitute [124]. The authors also investigated the age-dependence of engraftment in these mice and found that neonates can form 80% human cells, while one-week old animals can form 30% human cells, and two-week old animals can form 10% human cells at 8 weeks post implantation [116,124]. The preference for using neonates when reconstituting human cells is most likely due to a lesser developed murine thymus as compared to older mice or due to macrophages or neutrophils being less developed and conferring less resistance in newborns [116,124]. Using newborn Rag2^-/-^γ_c_^-/- ^animals, this study showed greater than 60% human cell engraftment in peripheral blood leukocytes and liver, and greater than 50% human cell engraftment in spleen and bone marrow [116,124]. This significant improvement in xenotransplantation in the Rag2^-/-^γ_c_^-/- ^model compared to the hu-SCID model provides a suitable environment to study infectious diseases and other maladies in a reliable small animal model.

The humanized Rag2^-/-^γ_c_^-/- ^scaffold is an ideal system to study HIV-1 pathogenesis due to the presence of an intact human immune system and its ability to support multi-lineage hematopoiesis. Two groups published the first evidence that this humanized mouse model can support a sustained HIV-1 infection [125,126]. The Baenziger *et al*. study utilized the Traggiai method of xenotransplantation into Rag2^-/-^γ_c_^-/- ^animals, and at 10–28 weeks of age the animals were infected i.p. with CCR5-tropic YU-2 or CXCR4-tropic NL4-3 HIV-1 viral strains [73,125]. Both HIV-1 strains were able to produce a chronic infection of up to 190 days as well as an initial acute burst phase of viral replication as detected by plasma viral RNA [125]. This group observed some strain-specificity in terms of CD4 T cell depletion and thymic infection. The CXCR4-tropic infected mice exhibited a marked depletion in CD4 T cell levels in the blood as compared to the CCR5-tropic strain, whereas the latter strain was able to infect the thymus of these animals almost exclusively. The Berges *et al*. study, which was focused on testing the permissiveness of this model to HIV-1 infection, was also performed using the xenotransplantation method of Traggiai *et al*. into conditioned neonatal BALB/c Rag2^-/-^γ_c_^-/- ^animals [126]. At 16 weeks post engraftment, thymic, splenic, and lymphoid tissue samples were taken from an animal and successfully infected with an X4-tropic NL4-3 HIV-1 reporter virus *ex-vivo *as measured by p24 ELISA. As the engrafted human cells were infectable, an *in vivo *infection was subsequently performed i.p. with HIV-1 X4-tropic NL4-3 or R5-tropic BaL viruses and sufficient levels of viral DNA was detected in the blood at up to 30 weeks post infection. This infection model also exhibited CD4 T cell depletion in the animals, a characteristic of chronic HIV-1 infections in humans. This study proved that the Rag2^-/-^γ_c_^-/- ^humanized mouse model can support an active infection *in vivo *and provide characteristic symptoms of viremia as seen in humans. These two studies were confirmed by Zhang *et al*. who reported that CCR5 and CXCR4 are both expressed on the reconstituted human T cells and peripheral lymphoid organs of this humanized mouse model [127]. They also reported that the HIV-1 infection in these mice persists for at least 19 weeks and that this model can serve to recapitulate HIV-1 immunopathogenesis.

Once the HIV-1 humanized mouse model was established in Rag2^-/-^γ_c_^-/- ^animals, multiple studies have improved upon the model through increased robustness of infection and the generation of infectable human cells. These mice were found to display both the Treg phenotype and functions of regulatory CD4+CD25+ T cells *in vivo *[128]. Specifically, it was found that the Treg cells and their interaction with the FoxP3 transcription factor (CD4+FoxP3+ cells) allow for the preferential infection by HIV-1 in the Rag2^-/-^γ_c_^-/- ^animals. Gorantla *et al*. developed an alternative irradiation dosing of neonatal mice at the time of xenotransplantation [129]. They combined lower-dosage irradiation (400-cGy) with busulfan-mediated myeloablation (destruction of quiescent stem cells), to result in a stable chimerism. Additionally, they found that all components of the human immune system were present at 16 weeks of age; however, the maturation of the immune system was not functional until sometime between five and six months of age. In terms of functional HIV-1 infection and viremia in this study, a low dose of HIV-1 C1157 was able to sustain a stable infection for at least eight to ten weeks. Additionally, infection with HIV-1 ADA resulted in the expansion of CD8+ cells, activation of B cells, and physical changes in the lymph nodes, similar to what occurs in human HIV-1 patients. A study by An *et al*. recently described similar successes in infecting the Rag2^-/-^γ_c_^-/- ^model with a reconstituted human immune system with R5 HIV-1 isolates [130]. Their results were in accordance with previous studies as they were able to detect virus in HIV-1 infected animals by co-culturing infected cells with non-infected cells *in vitro *as well as observing a decrease in the CD4+/CD8+ ratio as early as two weeks post-infection. With a low dose HIV-1 infection, this group detected B cell production of IgM and IgG, but were unable to detect an antibody response against HIV-1 antigens. Similarly, Van Duyne *et al*. showed successful infection of differentiated human CD45+ lymphocytes in their reconstituted Rag2^-/-^γ_c_^-/- ^model by *ex-vivo *infection with T-tropic or macrophage-tropic HIV-1 viruses [131] (Table 3). They also investigated the effect of inhibitors, i.e., AZT, Cyc202, and Tat peptide analogs on viral production in the Rag2^-/-^γ_c_^-/-^model. In HIV-1 infected and treated animals, viral DNA was still observed; however, there was a marked decrease in Gag DNA/RNA as compared to untreated animals. Importantly, this model is now being investigated for the efficiency of RNAi gene therapy against HIV-1 infection. Ter Brake *et al*. recently evaluated the inhibitory effect of a shRNA against Nef protein in HIV-1 infected Rag2^-/-^γ_c_^-/- ^animals [132]. The shRNA was transduced into the human hematopoietic stem cells prior to xenotransplantation, and the cells were allowed to differentiate into a normal percentage of cell subsets. Mature human CD4+ T cells were infected *ex vivo *with HIV-1, and a marked inhibition of viral replication was seen in the cells from the animals that received RNAi therapy. This study has very important implications for further experiments exploring RNAi as a therapeutic method.

The i.p. and i.v. methods of HIV-1 infection suffice for establishing a strong infection in these mouse models; however, they are not the natural routes of HIV-1 exposure in humans. Therefore, some recent studies have investigated the proficiency of these humanized mouse models in rectal and vaginal transmission. Berges *et al*. investigated the efficiency of transmission and infection of both R5 and X4 tropic HIV-1 viruses via both vaginal and rectal routes in the humanized Rag2^-/-^γ_c_^-/- ^mice [133]. Interestingly, not only did these humanized animals contain susceptible human cells in both the rectal and vaginal mucosa, these animals were readily infected with HIV-1 through intravaginal or intrarectal exposures [133]. A more efficient systemic infection was seen with the R5 mucosal infection as compared to the X4-HIV; however, more importantly, both viruses were able to infect the animals without mucosal abrasion or other means that are designed to make the animal more susceptible [133]. Interestingly, an opposing study was recently published where it was determined that the Rag2^-/-^γ_c_^-/- ^animals are not suitable for rectal transmission of HIV-1 [134]. Humanized Rag2^-/-^γ_c_^-/- ^animals were exposed rectally with both cell-free and cell-associated HIV-1 but the resulting viral load was negative as compared to animals infected via the traditional i.p. route [134]. Even upon various proinflammatory stimuli to increase the animals' susceptibility to HIV-1 infection, there was still a very low transmission rate due to low levels of human cellular engraftment in the gastro-intestinal associated tissues and cells [134].

## Humanized murine models for HTLV-1 infection

### HTLV-1 Pathogenesis

Another human retrovirus, Human T-cell leukemia virus type 1 (HTLV-1), has been identified as the causative agent of an aggressive form of adult T-cell leukemia (ATL) and HTLV-1-associated myelopathy/tropical spastic paraparesis (HAM/TSP) [135]. This virus has infected approximately 10 to 20 million individuals around the world, concentrating in several locations including, though not limited to, the islands of the Caribbean, the southwest area of Japan, and Central Africa [136,137]. Structurally, the virus is similar to other retroviruses in that it bears the *gag*, *pol*, and *env *genes, and long terminal repeats at the 5' and 3' ends [136]. One of the defining features of the virus is a region known as pX that encodes several regulatory proteins, including Tax, HBZ, and Rex [136,138,139]. In particular, Tax and HBZ have been associated with the clinical progression of disease in HTLV-1 infected individuals [136,140-143]. Successful transmission of the virus requires cell-to-cell contact and can be achieved through sexual intimacy, parenteral administration, or from a mother to infant via breast feeding. Expansion of the virus is achieved largely through proliferation of the infected cells, contributing to the clinical progression of disease [136]. Of the infected population, a small proportion will go on to develop ATL [137]. These individuals could experience a significant latency period of between 40 and 60 years. A diagnosis of ATL can be made using three criteria that include: lymphoid malignancy as proven through morphology and surface antigens, the demonstration of antibodies to HTLV-1 in the serum, and the use of Southern blot to shown integration of the HTLV-1 provirus [136,137]. The diagnosis of ATL can be further specified according to four unique subtypes with differing clinical characteristics that include smoldering, chronic, acute and lymphoma. The latter two, acute and lymphoma, are more aggressive and are typically associated with resistance to chemotherapy and subsequent poor outcomes. In the acute subtype, there may be an increase in ATL cell numbers, hepatosplenomegaly, lymphadenopathy, and skin lesions. The lymphoma subtype also demonstrates lympadenopathy throughout the body, although relatively few abnormal cells are seen in the peripheral blood. An individual diagnosed with one of these two subtypes will typically survive for an estimated duration of one year. The smoldering subtype demonstrates a low number of ATL cells with confirmed proviral integration in the peripheral blood. The chronic subtype has been shown to have a mildly elevated white blood cell count, as well as features similar to the acute type including hepatosplenomegaly, lymphadenopathy, and skin lesions. Often the chronic form will progress to acute or lymphoma with a mean survival time of about two years [144]. Cell-mediated immunity is involved in this disease process as evidenced by reports of co-infections with Mycobacterium, cytomegalovirus, and *Pneumocystis jiroveci *[136]. Another interesting feature of the disease is the development of hypercalcemia in 70% of ATL patients. Hypercalcemia is thought to be the result of an increase in osteoclast activity and associated resorption of bone tissue that is mediated through factors such as macrophage colony-stimulating factor (M-CSF) and macrophage inflammatory protein-1α (MIP-1α) [82,84,136,145]. Additionally, parathyroid hormone-related protein (PTHrP) has also been implicated as an important factor in the development of HTLV-1 infection and subsequent transformation of T-lymphocytes [146]. In addition to progression to ATL, HTLV-1 infection can also lead to the development of HAM/TSP in some individuals. Mechanistically, HTLV-1 associated tumor development and its associated symptoms may be explained in part through the actions of the viral genes Tax and HBZ [136,147,148]. Currently, therapeutic options for HTLV-1 infection include combination chemotherapy, allogenic stem cell transplantation, monoclonal antibodies, NF-κB-targeting, or the use of Zidovudine with IFN-α. Each of these approaches has limited success, and a reliable therapy remains to be found [136]. One other potential strategy is the dual use of cdk and NF-κB inhibitors [149]. The significance of this illness in combination with limited treatment options highlight the appeal of developing a successful small animal model for use in more clearly elucidating the pathogenesis of this infection as well as exploring therapeutic options.

### NOD/SCID β2-microglobulin^null ^and NOD/SCID IL2rγ^null^

As stated above, over the past 25 years science has seen the development of a variety of immunocompromised strains of mouse [69]. Most of these animals are the result of adjustments made to the 1983 CB17-*scid *mouse model. It was in this model that engraftment of human tissues was first observed in 1988. Ultimately, it was the development in the late 1990s of the NOD/SCID β2-microglobulin^null ^mouse as well as the NOD/SCID IL2rγ^null ^mouse that have proven to be truly valuable for investigations related to the HTLV-1 infection [68,69]. At that time the CB17-*scid *mouse model was improved upon by addressing the residual innate immune function through the creation of a mutation at the locus for β2 microglobulin (β2m); this mouse was subsequently known as the NOD/SCID β2-microglobulin^null ^mouse [64].

Investigation in the pathogenesis and potential treatment options related to HTLV-1 infection could be greatly enhanced by a useful small animal model. With the development of the humanized strains discussed above, the successful use of such a model became a realistic option. Early attempts to establish an HTLV-1 infection *in vivo *involved inoculation of the CB17-*scid *mouse model with PBLs or PBMCs from donors diagnosed with HTLV-1 infections. These experiments were promising although limited in success due to engraftment inefficiencies and poor detection of viral integration [150,151]. Feuer *et al*. took a different approach when they used the previously mentioned SCID-hu Thy/Liv model to compare the engraftment achieved with either infected human hematopoietic progenitor CD34+ cells or *in vitro *transformed HTLV-1 infected cell lines SLB-1 and MT-2 [145]. This group showed that not only could human hematopoietic progenitor cells be infected via co-cultivation with cell lines transformed with HTLV-1 and HTLV-2, but upon inoculation into SCID-hu Thy/Liv mice, infection could be detected in biopsies from the thy/liv organ. When the same model was challenged using only the transformed cell lines SLB-1 and MT-2, infection could be detected in biopsies from the thy/liv organ, although levels were not as impressive as those achieved with the hematopoietic progenitor cells. These results pointed to a role for hematopoietic cells in infection [145]. However, the limitations associated with the SCID-hu Thy/Liv model, especially the lack of systemic infection, caused investigators to continue to look to other models. An important development in the use of PBMCs for establishing such models was demonstrated by Liu *et al*. in their use of different HTLV-1 infected cell lines. This group noted that a higher level of engraftment could be achieved through the use of an HTLV-1 transformed cell line as opposed to cell lines that were immortalized through transfection, which did not produce lymphomas in NOD/SCID animals [152]. A subsequent investigation by Tanaka *et al*. in 2001 made use of the somewhat enhanced C3H/HEJ model and involved inoculation with MT-2 cells, a human T-cell line that produces HTLV-1 virus [153]. These colleagues were able to demonstrate integration of the virus as well as an apparent concentration of infected cells in lymphoid tissue. Miyazato *et al*. took the next step in 2006 when they designed an experiment utilizing the NOG mouse model [154]. Their investigation involved inoculation with PBMCs in order to establish a human system, followed by inoculation with the MT-2 cell line to allow for the required cell-to-cell transmission essential for HTLV-1 infection (Table 2, 3). Important findings included the detection of an increased proviral load in both CD4+ and CD8+ T cells. Additionally, they were able to demonstrate that prophylaxis with the reverse transcriptase inhibitors Tenofovir and Azidothymidine was successful in preventing new HTLV-1 infection in these animals. Takajo *et al*. was able to achieve similar results in 2007 when they also established HTLV-1 infection in NOG mice through the inoculation of PBMCs from HTLV-1 infected individuals [155]. Although the approach was somewhat different, the group confirmed that these animals could harbor HTLV-1 infection; and they demonstrated the presence of detectable viral integration.

The establishment of a successful murine HTLV-1 infection model quickly presented the opportunity for the investigation of treatment options. A report by Ohsugi *et al*. explored the use of the NF-κB inhibitor dehydroxymethylepoxyquinomycin (DHMEQ) as a therapeutic agent [156,157]. Ohsugi's group established a model for infection in the NOD/SCID β2-microglobulin^null ^mouse by sublethally irradiating 7 to 10 week old animals and injecting them with transformed HTLV-1 cell lines the following day. This investigation used the NOD/SCID β2-microglobulin^null ^mouse model to test the effectiveness of DHMEQ as a therapeutic option in HTLV-1 infection (Table 2, 3). Administration of DHMEQ showed increased survival and growth inhibition of ATL cells in animals that had been infected through inoculation with HTLV-1 producing cell lines. Another attempt to explore treatment options included a novel approach to detecting tumor growth. Shu *et al*. established a bioluminescent mouse model in the older CB17-*scid *model by infecting the animals with an ATLL cell line, RV-ATL, and a lentivirus harboring the luciferase gene [158]. These investigators were able to non-invasively measure the tumor growth and expansion which occurred in the recipient mice. Additionally, they tested both a bisphosphonate, zoledronic acid, and a protease inhibitor, PS-341. Both compounds demonstrated some level of success in reducing the development of tumors, as well as levels of parathyroid hormone-related protein (PTHrP) and macrophage inflammatory protein-1α (MIP-1α) which are both indicators of humoral hypercalcemia of malignancy (HHM), a complication observed in 60% of HTLV-1 infected individuals [158]. A recent publication by Nitta *et al*. utilized a mouse model with a defect in NF-κB inducing kinase (NIK) gene resulting in a phenotype of alymphoplasia (*aly*/*aly*) [159]. These investigators used this model to evaluate the importance of NIK for establishment of HTLV-1 infection and associated pathology. *Aly*/*aly *mice were compared with C57BL/6J and BALB/c mice. All animals were inoculated i.p. with MT-2 cells, and PCR was used to evaluate proviral load. *Aly*/*aly *animals demonstrated dramatically lower proviral loads, suggesting that NIK plays an essential role in HTLV-1 infection and could serve as a potential target for therapeutic intervention [159]. Chen *et al*. utilized the NOD/SCID mouse inoculated with an ATL cell line, MET-1, in their investigation of the use of a histone deacetylase inhibitor, depsipeptide, along with daclizumab as a therapeutic option in the murine HTLV-1 infection model [160]. Their results also showed promising findings, in that both depsipeptide and daclizumab alone and when used in combination, were able to increase the survival of the animals [160].

The evolution of immunocompromised murine models has enabled an increasingly successful investigation of the pathogenesis of HTLV-1 infection. As recently as the past three years, experimentation using these humanized mice has generated informed insights into the mechanisms associated with HTLV-1 infection. Such investigations utilizing a wide range of mouse models and varying infection techniques promise to mimic HTLV-1 infection in humans.

## Humanized mice and co-infection models

The humanized mouse models described above are clearly valuable research tools for the study of many kinds of disease. Additionally, a true model of HIV-1 infection in humans should not rule out the possibility and probability of co-infections amongst individuals. For example, Kaposi's sarcoma-associated herpesvirus (KSHV) or HHV-8 has been shown to proliferate in the SCID-hu Thy/Liv model both in the presence and absence of a concurrent HIV-1 infection [161]. Similarly, Human Herpesvirus 6 (HHV-6) is a herpesvirus that infects immunosupressed people as a result of HIV-1 infection. HHV-6 has been evaluated as a potential cofactor in the progression of HIV-1 when co-infected as modeled through the SCID-hu Thy/Liv system [162]. Both HIV-1 and HHV-6 are able to replicate in the engrafted humanized thymus in this model, and further studies can be done to evaluate the interplay between these two viruses *in vivo*. Finally, a unique interaction exists between HIV-1 infected individuals who are also infected with the protozoan *Toxoplasma gondii*. In immunocompetent individuals, the parasite persists in the CNS as an asymptomatic chronic infection; however, in the presence of an HIV-1 infection, and the subsequent decrease in CD4+ cells, the *T. gondii *infection can reactivate and cause a disease known as *Toxoplasma *encephalitis [163]. Alfonzo *et al*. investigated the co-infection of *T. gondii *in SCID mice humanized with PBMCs from HIV-1 infected patients who had been treated with HAART for at least one year [163]. Mice humanized with blood from patients undergoing HAART were more resistant to parasitic infection than those mice without any antiretroviral treatment. This study concluded that there is a partial immune reconsistitution against parasitic infection in HIV-1 infected individuals undergoing HAART therapy. Future studies should also look at the impact of genital HSV-2 infection on the acquisition of HIV-1 in humanized mice since epidemiological human data suggest that prior HSV-2 infection significantly enhances sexual transmission of HIV in women.

## Conclusion

Although still in its infancy, the field of humanized animal models holds enormous potential to grow as a primary research tool for human retroviral studies. Here we have reviewed the advances in this field over the past three decades, during which the technology and applications have grown exponentially. Future studies will most likely address how to increase the efficiency of mucosal infection in order to mimick the primary routes of HIV-1 transmission. The humanized mouse models also hold great promise for the development and testing of novel anti-retroviral therapies, bypassing the complications of larger animal (i.e., simian or human) studies. More generally, the humanized small animal model can benefit research in other human diseases such as cancers.

## Competing interests

The authors declare that they have no competing interests.

## Authors' contributions

Both RVD and CP contributed equally to this review.
